# Simultaneous integrated boost radiotherapy for bilateral breast: a treatment planning and dosimetric comparison for volumetric modulated arc and fixed field intensity modulated therapy

**DOI:** 10.1186/1748-717X-4-27

**Published:** 2009-07-24

**Authors:** Giorgia Nicolini, Alessandro Clivio, Antonella Fogliata, Eugenio Vanetti, Luca Cozzi

**Affiliations:** 1 Oncology Institute of Southern Switzerland, Medical Physics Unit, Bellinzona, Switzerland

## Abstract

**Purpose:**

A study was performed comparing dosimetric characteristics of volumetric modulated arcs (RapidArc, RA) and fixed field intensity modulated therapy (IMRT) on patients with bilateral breast carcinoma.

**Materials and methods:**

Plans for IMRT and RA, were optimised for 10 patients prescribing 50 Gy to the breast (PTVII, 2.0 Gy/fraction) and 60 Gy to the tumour bed (PTVI, 2.4 Gy/fraction). Objectives were: for PTVs V_90%_>95%, D_max_<107%; Mean lung dose MLD<15 Gy, V_20 Gy_<22%; heart involvement was to be minimised. The MU and delivery time measured treatment efficiency. Pre-treatment dosimetry was performed using EPID and a 2D-array based methods.

**Results:**

For PTVII minus PTVI, V_90% _was 97.8 ± 3.4% for RA and 94.0 ± 3.5% for IMRT (findings are reported as mean ± 1 standard deviation); D_5%_-D_95% _(homogeneity) was 7.3 ± 1.4 Gy (RA) and 11.0 ± 1.1 Gy (IMRT). Conformity index (V_95%_/V_PTVII_) was 1.10 ± 0.06 (RA) and 1.14 ± 0.09 (IMRT). MLD was <9.5 Gy for all cases on each lung, V_20 Gy _was 9.7 ± 1.3% (RA) and 12.8 ± 2.5% (IMRT) on left lung, similar for right lung. Mean dose to heart was 6.0 ± 2.7 Gy (RA) and 7.4 ± 2.5 Gy (IMRT). MU resulted in 796 ± 121 (RA) and 1398 ± 301 (IMRT); the average measured treatment time was 3.0 ± 0.1 minutes (RA) and 11.5 ± 2.0 (IMRT). From pre-treatment dosimetry, % of field area with γ <1 resulted 98.8 ± 1.3% and 99.1 ± 1.5% for RA and IMRT respectively with EPID and 99.1 ± 1.8% and 99.5 ± 1.3% with 2D-array (ΔD = 3% and DTA = 3 mm).

**Conclusion:**

RapidArc showed dosimetric improvements with respect to IMRT, delivery parameters confirmed its logistical advantages, pre-treatment dosimetry proved its reliability.

## Background

The aim of the present study was to investigate the potential clinical role of RapidArc, Varian Medical Systems (Palo Alto, CA), for a particularly complex and rare case of patients with synchronous bilateral breast carcinoma. In this study, RapidArc delivery is compared with ''conventional" fixed beam IMRT.

RapidArc falls into the general category of volumetric intensity modulated arc therapy (VMAT) [1-3] and it is a planning and delivery technique based on an investigation from K. Otto [4]. RapidArc and its precursor have been investigated previously for some other clinical cases [5-11], showing significant dosimetric improvements against other advanced techniques.

Breast radiation treatment with advanced techniques was investigated previously by our group and results [12,13] showed that in selected cases, IMRT is definitely beneficial compared to conventional conformal approaches.

The simultaneous integrated boost (SIB) fractionation strategy proposed in this study is justified by two rather general objectives: i) reduce the length of treatment to improve patient satisfaction and clinical throughput; ii) to assess dosimetric potentials of advanced techniques and planning capabilities.

Limited investigations on SIB in breast and on bilateral breast irradiation are available in literature. Hurkmans et al, Singla et al. and van der Laan et al. [14-16] analysed this option proposing different schemes: 28x(1.81+2.3) Gy or 31x(1.66+ 2.38) Gy for remaining breast and tumour bed targets. In all cases, the SIB plans with IMRT proved superior quality compared to sequential treatments and authors [15] proposed to consider SIB as standard treatment. In the present study it was opted to propose a further acceleration in the fractionation planning for 25 fractions (to keep treatment time limited to five weeks) of 2.0 Gy to entire breast with a simultaneous integrated boost of 2.4 to the tumour bed. This fractionation has yet to be proven to be clinically acceptable; however, it does not impact the significance of comparative results.

Jobsen et al, Skowronek et al. and Yamauchi et al [17-19] investigated the radiation therapy options as well as the prognostic and incidence of synchronous or metachronous bilateral breast cancer. These studies demonstrated the technical feasibility of bilateral irradiation with conventional techniques. The incidence of synchronous bilateral breast cancer is quite low of the order of 1.5% (18 patients over 1705 in the Jobsen study) associated to a higher incidence of distant metastases and a worse disease free survival.

Although rare, synchronous bilateral breast irradiation is a complex situation where the concomitant involvement of both lungs and heart and the huge treated volume is a particular challenge.

To minimise patient discomfort, it is advisable to investigate also potentials of fast delivery techniques. While standard treatment times are of the order of 15 minutes, individual patient compliance with immobilisation devices during 20–25 minute treatments may be compromised because of their disease status or because of involuntary factors (e.g. coughing induced by swallowing in the supine position). The drawback of some IMRT techniques, is the extended time needed to deliver one fraction, mostly because of the usage of multiple fields and high number of MUs.

Purpose of the present investigation was: i) to assess, for a relatively rare pathology, the quality of two advanced treatment techniques in terms of expected dose distributions and pre-treatment dosimetric verification; ii) to quantify the differences between the two solutions and iii) to appraise logistic aspects as treatment efficiency. The latter point does not necessarily apply to rare pathologies but is of interest since, for RapidArc, multiple arcs were applied instead of single arcs and knowledge of the impact of arc multiplicity on treatment efficiency is still limited.

## Methods and patients

### Patient selection and planning objectives

Anonymized CT data for a cohort of ten consecutive patients treated for bilateral breast carcinoma after breast conserving surgery, were used for the study. All patients had ductal or lobular carcinoma in different quadrants, stage T1(b or c), N0M0 and underwent breast conserving surgery (lumpectomy); median age 69 (range: 67–85). CT scans were acquired with 5 mm adjacent slice thickness in free breathing mode. Scan extension included the entire lung volume and reached, cranially, the supra-clavicular level. In terms of lung volumes and relative positions of lungs, heart and target volumes, free breathing can be considered as a first order surrogate of a mid-ventilation phase of the breathing cycle. Treatment was planned with patients in the supine position. The main organs at risk (OAR) considered were lungs and heart. Lung mean volumes were: 1080 ± 165 cm^3 ^(left) 1390 ± 267 cm^3 ^(right); heart mean volume was: 377 ± 110 cm^3^. The healthy tissue was defined as the patient's volume covered by the CT scan minus the envelope of the various planning target volumes (PTV).

Four target volumes were defined by radiation oncologists: CTVII (left and right) was the clinical target volume encompassing the entire breast while CTVI (left and right) was the boost volume defined by the tumour bed defined as the lumpectomy volume. PTVII and PTVI (left and right) were obtained with expansion of 8 mm in all directions except toward skin. PTVs were restricted to the skin cropping at 5 mm from surface and to exclude the ribs. The mean volumes were: PTVII: 612 ± 316 cm^3 ^(left), 679 ± 318 cm^3 ^(right), PTVI: 47 ± 16 cm^3 ^(left), 59 ± 29 cm^3 ^(right). Target definition for CTVI was performed without help of surgical clips, not implanted in the patients. This procedure is acknowledged to be suboptimal and, in clinical practice, it is advisable to use these or similar tools to improve this volume definition and to minimise risk of geographical misses.

Dose prescription was according to a Simultaneous Integrated Boost (SIB) scheme with 50 Gy (2 Gy/fraction) to PTVII and 60 Gy (2.4 Gy/fraction) to PTVI. This fractionation was assumed in absence of a general consensus in literature on SIB strategy in breast as discussed in the introduction. All plans were normalised to the mean dose of the total PTVII minus PTVI (PTVII-PTVI) volume (i.e. left plus right) as common practice for intensity modulated plans and in agreement to forthcoming ICRU recommendations.

For all PTVs, plans aimed to achieve at least 95% of the PTV receiving more than 90% of the prescribed dose and, for PTVI, a maximum lower than 107% while keeping the mean dose of each PTV as close as possible to the corresponding prescription. Given the PTV definitions and given the decision to avoid usage of bolus in this theoretical study (in principle applicable to both IMRT and RapidArc), the objectives on PTVII minimum dose are expected to be difficult to respect. To prevent skin toxicity, bolus usage should be minimised or, at least, applied on alternate days and was considered as a potential confounding factor in the study. For lungs, given the bilateral involvement, although conventional objectives were considered as acceptable (i.e. mean lung dose MLD<15 Gy and volume receiving at least 20 Gy V_20 Gy_<22% [20-22]), plans were designed to maximise lung sparing. Similarly for heart, the planning strategy was to minimise mean and maximum doses.

### Planning techniques

Two sets of plans were compared in this study, all designed by the same planner on the Varian Eclipse treatment planning system (TPS) (version 8.6.10) with 6 MV photon beams from a Varian Clinac equipped with a Millennium Multileaf Collimator (MLC) with 120 leaves (spatial resolution of 5 mm at isocentre for the central 20 cm and of 10 mm in the outer 2 × 10 cm, a maximum leaf speed of 2.5 cm/s and a leaf transmission of 1.8%). Plans for RapidArc were optimised selecting a maximum DR of 600 MU/min and a fixed DR of 600 MU/min was selected for IMRT.

The Anisotropic Analytical Algorithm (AAA) photon dose calculation algorithm was used for all cases [23,24]. The dose calculation grid was set to 2.5 mm.

#### IMRT

The dynamic sliding window method with fixed gantry beams was used [25,26].

**Figure 1:**
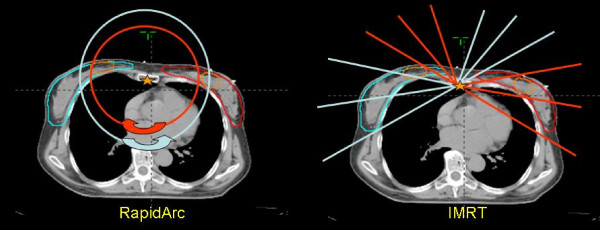
**Beam arrangements, isocentre position and targets localization for IMRT and RapidArc**. For RapidArc, two arcs, rotating in opposite directions, are delivered in sequence, each arc aiming to geometrically cover primarily either left (red arc) or right targets (blue arc). For IMRT a similar approach was followed. Six fixed gantry field aimed to geometrically cover left targets (red lines showing the central beam axes) and the other 6 (blue lines) the right targets.

Plans were optimised for a mono-isocentric approach with the single isocentre located medially under the sternum. Twelve beams with fixed jaws settings were applied, starting from 120° and equally-spaced every 20° (excluding the 0° entrance). 6 beams were shaped to cover primarily the left breast (120°, 100°, 80°, 340°, 320°, 300°) and 6 the right breast (60°, 40°, 20°, 280°, 260°, 240°) according to the pattern shown in figure 1. Beam angles were selected in order to i) remain within the limit of 5–7 beams per target as described in [12]; ii) avoid posterior entrance to enhance preservation of lungs and heart; iii) mimic a sort of tangential distribution of beams. All beams were coplanar with collimator angle set to 0° as per institutional standards and because on fixed gantry fluence based IMRT this has a marginal impact on modulation capability. No bolus and no fluence expansion outside body (skin flash) were applied to IMRT (and to RapidArc). A high smoothing factor was applied during optimisation (with the same priority of the highest priority used for dose volume objectives) to minimise the MU/Gy from IMRT. The beam arrangement chosen for this study resulted, among other investigated for the purpose, the best trade-off between target coverage, OARs sparing and practical feasibility. It is possible that other arrangements could generate better plans but were not identified for this study.

#### RapidArc (RA)

RapidArc uses continuous variation of the instantaneous dose rate (DR), MLC leaf positions and gantry rotational speed to optimise the dose distribution. Details about RapidArc optimisation process have been published elsewhere and readers are referred to original publications for details [5,6]. To minimise the contribution of tongue and groove effect during the arc rotation and to benefit from leaves trajectories non-coplanar with respect to patient's axis, the collimator rotation in RapidArc remains fixed to a value different from zero [27]. In the present study collimator was rotated to ~10°–30° depending on the patient. Plans were optimised with two arcs of 360° each. The first arc, rotating clockwise, was incident primarily on the right breast, the second arc, rotating counter-clockwise, was incident on the left breast as depicted in figure 1.

The same isocentre was used for IMRT and RapidArc plans. In both cases, all fields or arcs were simultaneously optimised to generate the desired dose distributions on all targets.

Both RapidArc and IMRT plans were optimised using exactly the same dose volume objectives and constraints and with the same prioritisation of organs. Lung sparing had higher priority than heart or normal tissue.

### Pre-treatment Quality Assurance dosimetric measurements

To assess delivery quality and the agreement between calculations and treatment, standardised pre-treatment quality assurance dosimetric measurements were performed verifying each individual field or arc. Two dosimetry methods and detectors were applied:

a) the GLAaS method. This method has been investigated widely [28,29]. In brief, it consists in measurements performed with the amorphous silicon portal imager Portal Vision PV-aS1000, attached to the treatment linac, with a calibration and processing method converting raw data into absorbed doses at depth of maximum (1.5 cm in this case). GLAaS has been already tested for RapidArc delivery [28] and is the reference dosimetry tool in our centre for pre-treatment verifications. With GLAaS no additional phantom has to be used and, for RapidArc, the detector rotates together with the gantry generating a sort of collapsed or composite planar dose distribution. Spatial resolution of the GLAaS measurements is 0.392 mm in x and y (PV-aS1000 pixel size).

b) The PTW-729 method. The 2D ion chamber array from PTW (the 729 model) was used. For IMRT verifications the detector was positioned at isocentre with an additional build up of 7 mm equivalent solid water (to reach an equivalent measuring depth of 1.5 cm). For RapidArc verification, the Octavius phantom developed by PTW for rotational therapy verification was used. In this case, the detector remains fixed on the treatment couch during delivery and therefore the measurement generates a planar dose different from the GLAaS one but similarly of composite nature. To compare measurements and calculations, the Octavius-729 system was CT scanned and the RapidArc plans were recalculated on this CT dataset. Detector was positioned at isocentre. Spatial resolution of PTW-729 measurements is coarser than with the GLAaS being the detector made by square ion chambers with 5 × 5 mm^2 ^surface and inter-centre spacing of 10 mm.

### Evaluation tools

Evaluation of plans was based on Dose-Volume Histogram (DVH) analysis. For PTV, the values of D_98% _and D_2% _(dose received by the 98, and 2% of the volume) were defined as metrics for minimum and maximum doses. Also V_90% _V_95% _V_107% _and V_110% _(the volumes receiving at least 90%, 95%, 107% or 110% of the prescribed dose) were reported. The homogeneity of the dose distribution, was measured by D_5%_-D_95%_. The lower this value, the better is the dose homogeneity. Equivalent Uniform Dose (EUD) was computed with α = 0.15 Gy^-1^, α/β = 2.8 Gy [30].

Conformity Index, CI_90% _and CI_95%_, ratio between the patient volume receiving at least 90% (95%) of the prescribed dose and the volume of the total PTVII, measured the conformity of the dose distribution. To account for the spillage of prescription dose in the healthy tissue, the External Volume Index (EI) was defined as V_D_/V_PTVII _where V_PTVII _is the volume of the total PTVII and V_D _is the volume of healthy tissue receiving more than 50 Gy.

For OARs, the analysis included the mean dose, the maximum dose expressed as D_2% _and a set of V_XGy _(OAR volume receiving at least × Gy) depending upon the organ. Normal Tissue Complication Probability (NTCP) was computed using the relative seriality model of Källmann *et al. *[31,32]. The following values for the model's parameters were used: γ = 1.7, *s *= 0.03, D_50 _= 26.0 Gy for pneumonitis and γ = 3.0, *s *= 0.2, D_50 _= 49.0 Gy for pericarditis, where *s *represents the degree of seriality for the organ, γ is the dose-response steepness index and D_50 _is the dose to the whole organ to induce NTCP = 50%.

For Healthy Tissue, the integral dose, "DoseInt" was defined as the integral of the absorbed dose extended to over all voxels excluding those within the target volume (DoseInt dimensions are Gy*cm^3^). This was reported together with the observed mean dose, V_3 Gy _and V_10 Gy_.

Average cumulative DVH for PTV, OARs and healthy tissue, were built from the individual DVHs for qualitative visualisation of results. These histograms were obtained by averaging the corresponding volumes over the whole patient's cohort for each dose bin of 0.05 Gy.

Delivery parameters were recorded in terms of MU per fraction, mean dose rate, MU/degree, beam on time and treatment time (defined as beam-on plus machine programming and setting time and excluding patient positioning and imaging procedures).

Pre-treatment quality assurance results were summarised in terms of the Gamma Agreement Index, GAI, scoring the percentage of modulated area fulfilling the γ index criteria [33] (computed with 2 and 3% and 2 and 3 mm thresholds). The software utilised to analyse dosimetric data were either the GLAaS package developed by authors or the Verisoft (version 4.0) from PTW. In both cases, γ computation was performed using the maximum dose value in the calculated matrix as normalisation for dose difference evaluation. In both cases, γ was computed with respect to the measured points and therefore was based on a maximum of 729 entries in the PTW case and on a maximum of 1024 × 768 pixels in the GLAaS case (both reduced according to the modulated field area seen by the detector). Pre-treatment dosimetry was considered satisfactory if GAI exceeded 95%.

The Wilcoxon matched-paired signed-rank test was used to compare the results. The threshold for statistical significance was *p *= 0.05. All statistical tests were two-sided.

## Results

**Figure 2:**
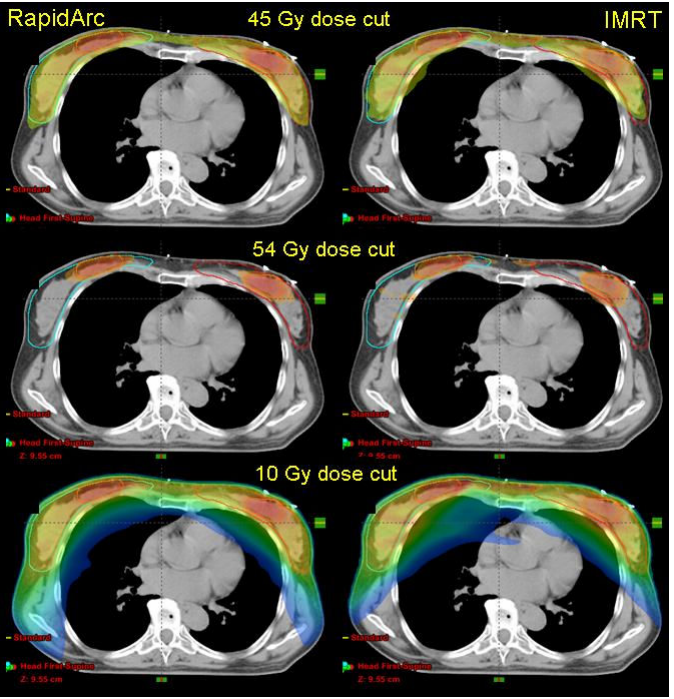
**Example of dose distributions on axial views for one case**. Color wash thresholds were set to 45 or 54 Gy, 95% of the respective dose prescriptions to PTVII and PTVI, and to 10 Gy to represent the total dose bath.

**Figure 3:**
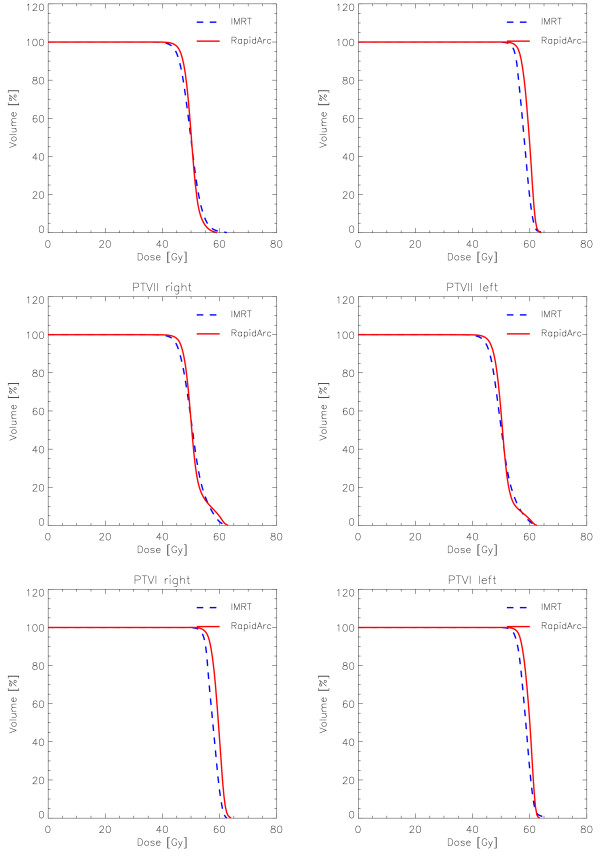
**Mean DVHs (averaged over the 10 patients) for the various PTVs**.

**Figure 4:**
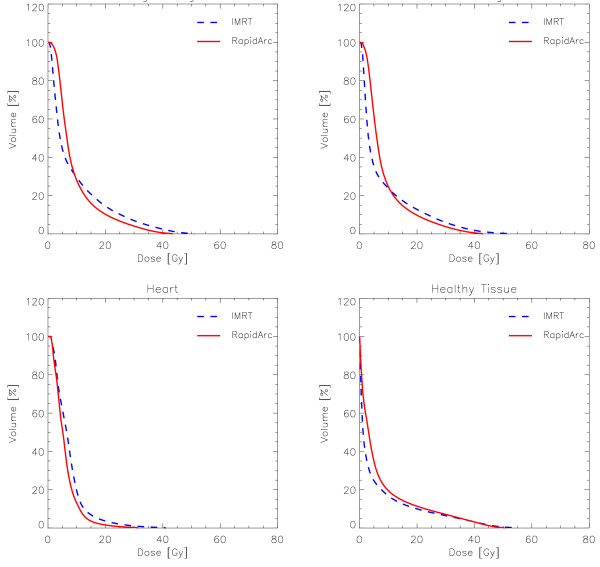
**Mean DVHs (averaged over the 10 patients) of the left and right lungs, heart and healthy tissue (total body volume in the CT set minus the total PTVII)**.

Dose distributions are shown for one example in Figure 2 for axial views and three dose cuts (45 Gy, 90% of PTVII prescription; 54 Gy, 90% of PTVI prescription, and 10 Gy). Figures 3 and 4 show the average DVH for all the PTVs, lungs, heart and healthy tissue. Tables 1, 2 and 3 summarise numerical findings from DVH, delivery and pre-treatment dosimetry analyses. Data are presented as averages over the investigated patients and errors indicated inter-patient variability at 1 standard deviation level.

**Table 1:** Summary of DVH based analysis for the PTVII and PTVI

	**PTVII-PTVI (left and right)**			**PTVI (left and right)**		
	**RapidArc**	**IMRT**	**p**	**RapidArc**	**IMRT**	**p**
**Mean (Gy)**	50	50	-	59.6 ± 0.9	58.0 ± 0.9	0.004
**EUD (Gy)**	48.5 ± 0.6	47.6 ± 0.8	0.003	58.9 ± 0.7	57.7 ± 1.0	0.004
**D**_5%_**-D**_95%_**(Gy)**	7.3 ± 1.4	11.0 ± 1.1	0.004	3.4 ± 0.7	5.8 ± 0.9	0.50
**D**_2%_**(Gy)**	55.8 ± 1.1	57.2 ± 1.6	0.004	62.3 ± 0.8	61.9 ± 1.4	0.145
**D**_98%_**(Gy)**	45.1 ± 1.2	43.4 ± 1.3	0.004	55.8 ± 1.3	54.1 ± 1.3	0.035
**V**_90%_**(%)**	97.8 ± 2.4	94.0 ± 3.5	0.004	99.3 ± 1.5	97.5 ± 3.0	0.035
**V**_107%_**(%)**	8.3 ± 5.1	14.0 ± 5.3	0.004	0.0 ± 0.0	0.0 ± 0.0	0.004
**V**_110%_**(%)**	4.0 ± 2.3	8.5 ± 4.3	0.035	0.0 ± 0.0	0.0 ± 0.0	-

	**PTVII left**			**PTVII right**		
	**RapidArc**	**IMRT**	**p**	**RapidArc**	**IMRT**	**p**

**Mean (Gy)**	51.0 ± 0.3	50.8 ± 0.3	0.004	50.9 ± 0.8	50.5 ± 0.6	0.145
**EUD (Gy)**	48.8 ± 0.7	47.7 ± 0.9	0.002	48.7 ± 0.6	48.0 ± 0.7	0.06
**D**_5%_**-D**_95%_**(Gy)**	9.7 ± 0.9	14.2 ± 1.4	0.363	9.8 ± 1.8	10.5 ± 1.3	0.363
**D**_2%_**(Gy)**	60.6 ± 1.3	59.7 ± 1.4	0.363	60.2 ± 1.6	59.7 ± 1.7	0.145
**D**_98%_**(Gy)**	45.2 ± 1.5	43.6 ± 1.6	0.004	45.2 ± 1.3	43.5 ± 1.0	0.004
**V**_90%_**(%)**	98.0 ± 2.1	94.7 ± 3.4	0.004	95.7 ± 2.4	93.3 ± 2.4	0.004
**V**_110%_**(%)**	10.4 ± 2.6	14.2 ± 5.5	0.035	10.1 ± 5.2	13.4 ± 5.6	0.004

	**PTVI left**			**PTVI right**		
	**RapidArc**	**IMRT**	**p**	**RapidArc**	**IMRT**	**p**

**Mean (Gy)**	59.4 ± 1.1	58.6 ± 1.4	0.06	59.7 ± 0.8	58.5 ± 0.6	0.004
**EUD (Gy)**	59.8 ± 0.5	58.3 ± 0.7	0.03	59.7 ± 0.9	57.4 ± 1.3	0.005
**D**_5%_**-D**_95%_**(Gy)**	5.8 ± 0.6	7.4 ± 1.0	0.5	4.3 ± 1.0	5.7 ± 1.5	0.145
**D**_2%_**(Gy)**	62.1 ± 1.2	60.3 ± 1.8	0.5	62.2 ± 0.6	62.2 ± 1.6	0.145
**D**_98%_**(Gy)**	55.7 ± 1.4	54.0 ± 1.2	0.004	56.0 ± 1.3	54.3 ± 1.3	0.035
**V**_90%_**(%)**	99.5 ± 0.6	98.5 ± 2.0	0.035	99.5 ± 0.8	97.9 ± 2.5	0.035
**V**_110%_**(%)**	0.0 ± 0.0	0.2 ± 0.5	0.5	0.0 ± 0.0	0.0 ± 0.0	-

Data are reported as mean ± 1 standard deviation computed over the cohort of 10 patients.

D_x% _= dose received by the x% of the volume; V_x% _= volume receiving at least x% of the prescribed dose; EUD = Equivalent Uniform Dose.

* p < 0.05; ** p < 0.01

**Table 2:** Summary of DVH based analysis for OARs and healthy tissue

	**Left Lung**			**Right Lung**		
	**RapidArc**	**IMRT**	**p**	**RapidArc**	**IMRT**	**p**
**Mean (Gy)**	8.7 ± 1.0	7.8 ± 0.9	0.15	9.4 ± 1.2	9.1 ± 1.4	0.4
**D**_2%_**(Gy)**	34.3 ± 2.3	39.4 ± 4.1	0.04	37.9 ± 1.7	44.8 ± 2.5	0.004
**V**_5 Gy_**(%)**	58.7 ± 18.9	35.3 ± 3.9	0.004	58.3 ± 18.9	44.4. ± 7.8	0.004
**V**_20 Gy_**(%)**	9.7 ± 1.3	12.8 ± 2.5	0.04	10.3 ± 1.4	14.5 ± 4.0	0.004
**V**_45 Gy_**(%)**	0.1 ± 0.2	0.9 ± 0.7	0.04	0.1 ± 0.1	0.9 ± 0.5	0.004
**NTCP (%)**	<0.1	<0.1	0.3	<0.1	<0.1	0.2
			
	**Heart**					
	**RapidArc**	**IMRT**	**p**			
			
**Mean (Gy)**	6.0 ± 2.7	7.4 ± 2.5	0.14			
**D**_2%_**(Gy)**	17.0 ± 6.6	24.4 ± 7.1	0.04			
**V**_10 Gy_**(%)**	13.1 ± 14.1	19.5 ± 13.3	0.04			
**V**_45 Gy_**(%)**	0.0 ± 0.0	0.1 ± 0.1	0.12			
**NTCP (%)**	<0.1	<0.1	0.25			
			
	**Healthy Tissue**					
	**RapidArc**	**IMRT**	**p**			
			
**Mean (Gy)**	7.1 ± 0.3	5.0 ± 0.4	0.03			
**V**_3 Gy_**(%)**	50.1 ± 8.7	33.5 ± 6.1	0.03			
**V**_10 Gy_**(%)**	20.5 ± 3.3	18.3 ± 2.7	0.19			
**CI**_90_	1.19 ± 0.07	1.20 ± 0.07	0.19			
**CI**_95_	1.10 ± 0.06	1.14 ± 0.09	0.19			
**EI (%)**	3.7 ± 1.9	8.7 ± 2.9	0.19			
**DoseInt (Gycm**^-3^**10**^5^**)**	1.40 ± 0.36	1.15 ± 0.27	0.03			

Data are reported as mean ± 1 standard deviation computed over the cohort of 10 patients.

D_x% _= dose received by the x% of the volume; V_xGy _= volume receiving at least × Gy; CI = ratio between the patient volume receiving at least 90% and 95% of the prescribed dose and the volume of the total PTVII; EI = V_D_/V_PTVII _where V_PTVII _is the volume of the total PTVII and V_D _is the volume of healthy tissue receiving more than 50 Gy; DoseInt = integral of the absorbed dose extended to over all voxels excluding those within the target volume; NTCP = Normal Tissue Control Probability with the relative seriality model.

* p < 0.05; ** p < 0.01

**Table 3:** Summary of delivery parameters and pre-treatment dosimetric tests

**Delivery parameters**					
**2 Gy/fraction**	**RapidArc**		**IMRT**		

**Monitor Units (MU)**	796 ± 121		1398 ± 301		p < 0.01
**MeanDose Rate (MU/min)**	378 ± 46		600 fixed		-
**Mean MU/**^°^	1.1 ± 0.2		-		-
**Beam on time (min)**	2.30 ± 0.01		2.23 ± 0.40		p = 0.14
**Treatment time (min)**	3.00 ± 0.08		11.45 ± 2.05		p < 0.01

**Pre-treatment Quality Assurance**					

	**RapidArc**		**IMRT**		
	**GLAaS**	**PTW729**	**GLAaS**	**PTW729**	

**GAI (3% 3 mm) (%)**	98.8 ± 1.3 ^a^	98.8 ± 1.3 ^a^	99.1 ± 1.5 ^a^	99.5 ± 1.3 ^a^	
**GAI (2% 3 mm) (%)**	97.0 ± 2.8 ^a^	97.0 ± 2.8 ^a^	97.2 ± 4.3 ^a^	98.4 ± 3.0 ^a^	
**GAI (2% 2 mm) (%)**	93.0 ± 5.0	93.0 ± 5.0	94.4 ± 4.3	96.3 ± 4.3	

Data are reported as mean ± 1 standard deviation computed over the cohort of 10 patients.

GAI is the Gamma Agreement Index, percentage of field area of percentage of measured points fulfilling the criteria γ <1.

* p < 0.05; ** p < 0.01; ^a ^p < 0.05 w.r.t. GAI = 95%

**Figure 5:**
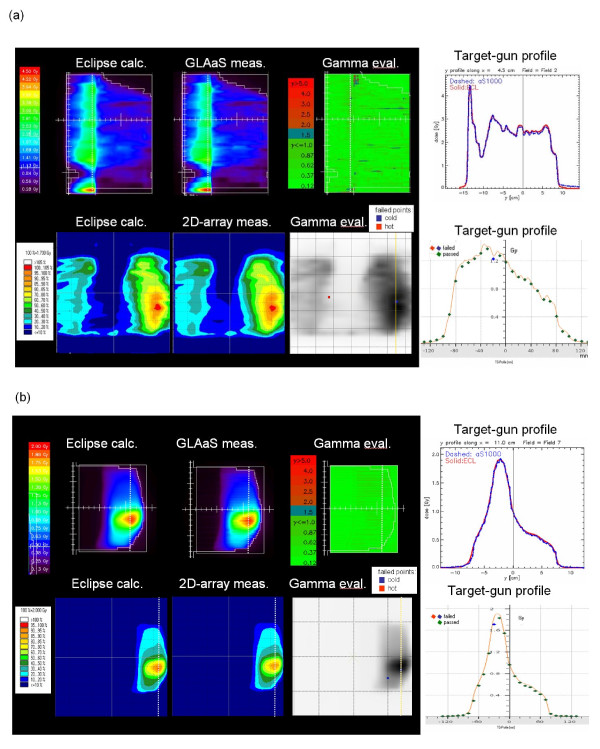
**Example of pre-treatment dosimetric measurements with the GLAaS method and with the PTW-729: a) IMRT, b) RapidArc**. In each figure it is shown: i) 2D dose maps calculated by Eclipse and measured at Linac. Planes shown are at 15 mm depth in water (GLAaS) or at isocentre PTW-729); ii) the 2D γ map for GLAaS or the overlay between the calculated 2D dose map and the detector points violating the threshold γ <1; iii) dose profiles along the dashed line shown in the 2D maps, comparing measure and calculation. In the case PTW-729 calculation is the solid line while measurements are the points.

Figure 5 shows the results from pre-treatment quality assurance for one IMRT field and one RapidArc arc from the two dosimetric methods applied in the study. Shown are the planar dose maps at isocentre (2D-array) and 1.5 cm depth (GLAaS) computed from the measured data, the 2D γ map from the comparison against corresponding calculations and a profile along the y direction. Summary of numerical findings is reported in table 3 together with the results from other delivery parameters.

### Target coverage and dose homogeneity

Data in the tables are reported for the total target volumes, combining left and right sides, as well as for the separated targets referring the DVH for each PTV to the dose prescribed (e.g. for PTVI 100% = 60 Gy). In general, RapidArc and IMRT achieved similar results. IMRT resulted in a slight under dose to the boost volume while RapidArc better respected the dose prescription. RapidArc reduced D_5%_-D_95% _of more than 3.5 Gy to bilateral PTVII-PTVI compared to IMRT. Similarly, homogeneity was improved in the case of PTVI. A reduction of over dosages in the PTVII-PTVI volume for RapidArc was also observed compared to IMRT. RapidArc showed also an improvement in target coverage: for PTVII-PTVI or PTVI (V_90%_). EUD improved of ~1 Gy on PTVII-PTVI and PTVI for RapidArc compared to IMRT.

Equivalent findings were obtained analysing each target separately, proving no differences in the optimisation of dose distributions between the right or left sides of the patient.

### Organs at risk

High sparing of lungs was achieved with both techniques. The observed differences on MLD are not statistically significant. At medium to high levels, RapidArc proved to be slightly superior to IMRT. At low dose levels, e.g. V_5 Gy_, IMRT was better than RapidArc.

For the heart, RapidArc results were superior to IMRT at all dose ranges.

### Healthy tissue

The mean and the integral dose were found to be higher with RA with respect to IMRT due to a higher contribution at low dose levels (e.g. V_3 Gy_). On the contrary, RapidArc was better than IMRT in lowering the high dose levels for soft tissues of interest (e.g., to improve cosmetic results). RapidArc reduced EI compared to IMRT.

### Delivery parameters

The ratio between number of MU per fraction of 2 Gy (2.4 Gy on the boost volumes) resulted to be MU_IMRT_/MU_RA _= 1.76. The average dose rate for RA deliveries resulted ~60% of the fixed dose rate applied to IMRT deliveries and, upfront to a statistically not significant difference in beam on time, the treatment time was nearly 74% less for RapidArc compared to IMRT. This is mostly due to the need to reprogram the linac between fixed gantry beams, rotate the gantry from one position to the next and to deliver split fields (since with dynamic sliding window IMRT, main jaws are fixed during delivery, fields exceeding ~14 cm in width are split in two or three carriage groups to compensate for this hardware feature; in the present study, in average three-four beams per patients were split). For RapidArc, all individual arcs could be delivered between 83 to 85 seconds of beam on time.

### Pre-treatment dosimetric measurements

A summary of findings is reported in table 3 for the various combinations of thresholds. Concerning GLAaS, the dosimetric agreement between calculation and delivery resulted to be highly satisfactory. Similarly high quality results were obtained with the PTW-729 system.

## Discussion and conclusion

The planning case selected for this investigation is highly demanding because of several factors: i) total target volumes are huge (about 1400 cm^3^), ii) bilateral involvement of lungs and of heart requires tight avoidance capabilities, iii) treatment shall be technically easy to administer and as fast as possible.

The first objective was to prove the possibility to create treatment plans of high quality with one single isocentre located in the mid-line of the sternum to allow easy and safe management of patients. This was achieved nicely by both techniques but required the application of 12 beams with IMRT and 2 independent arcs with RapidArc. Concerning RapidArc, due to their simultaneous optimisation, each of the two arcs contributes to the dose at both sides even though each is geometrically mainly incident on either the left or right target only. Some under-dosage of PTVs was expected and due to the extension of the targets till the proximity of patient's surface. To eliminate this feature it would be possible to further crop PTV inside the body [14] or to add a bolus in the optimisation and calculation phases. Both approaches were not followed to stick with institutional standards and to generate plans under the most restrictive conditions. Nevertheless RapidArc respected the planning objective on V_90% _while IMRT presented a minor violation. Concerning IMRT plans, the decision to avoid bolus in the optimisation does not increase the risk of excessive skin toxicity because in Eclipse fluence matrices are normally generated without un-necessarily high fluence in those beamlets impinging tangentially to the skin to compensate for low doses in the build-up region. In addition, the usage of high smoothing factors further reduces the presence of small hot (or cold) spots in the fluence matrices as well as reduces high frequency changes in the intensity of the fluence beamlets.

The quality of delivered doses compared to the computed was assessed with pre-treatment dosimetry. RapidArc and IMRT proved to be equivalent using two totally independent methods of verification. The excellent quality of dosimetric results guarantees about the safety of the newer technique. Sensitivity of the RapidArc technique to tighter thresholds was investigated and proved to be highly satisfactory with both the GLAaS and PTW-729 methods. The quality of GLAaS based measurement for RapidArc is confirmed in other studies with different detectors [10,34] where either gafchromic films or other 2D systems were used and GAI or equivalent metrics exceeded 95% as well. This consistency suggests also the limited relevance of the fact that with GLAaS dosimetry the EPID detector rotates together with the gantry although it would not allow detecting potential mismatches between planned and actual positions.

The second objective was to quantify quality of dose distributions and potential differences between RapidArc and IMRT. The data shown here suggest that both techniques are satisfactory. RapidArc offers some improvement on target coverage, homogeneity and lung and heart sparing. Concerning V_5 Gy_, as can be derived from the graphs in figure 4, the steep gradient of DVHs in the low dose range, makes the absolute validity of numbers questionable since small deviations in dose thresholds corresponds to huge variations in volumes. The clinical relevance of the observed differences cannot be drawn simply from a planning study with limited statistical power and appropriate trials should be performed.

The third objective was to assess treatment efficiency. Pure beam on time was equivalent between IMRT and RapidArc. Total treatment time was assessed measuring the time needed from loading the first beam to completing the last beam, i.e. accounting for all technical delivery aspects but excluding patient positioning and pre-treatment imaging procedures to verify patient positioning that should be equivalent between techniques. RapidArc treatment times were 74% shorter than IMRT implying a reduction of the risk of intra-fractional movements. The number of split fields with single isocentre IMRT is not higher than a corresponding value if double isocentre is used, since the field width is dominated by the PTVII width and field sizes are similar with both approaches thanks to the usage of asymmetric jaws settings. The present comparison refers anyway to a specific implementation of fixed beam IMRT, the Dynamic Sliding Window. Different approaches, e.g. based on direct aperture, or with fewer gantry angles or with few segments or avoiding split fields, could improve efficiency of IMRT.

Specific to this investigation, it is the role of motion management and breath control. In the study and in clinical practice for similar patients, no breast immobilisation system is applied, but only an arm support is used. Immobilisation could be advisable in the case of large breasts but, at present, no satisfactory solution was found at our institute. From the dosimetric point of view, avoidance of lungs and of heart in breast irradiation was proven to be significantly improved [35] if irradiation is performed with gated delivery in the deep inspiration phase. At the current stage, breath control is available for conventional IMRT but not yet for RapidArc. Nevertheless, the mid-ventilation phase could be an adequate surrogate of breath control since, statistically, it is the phase where targets can be ''seen" by static beams for the longest time provided adequate margins are defined. In the present study, the CT dataset used can be considered as average mid-ventilation phase partially solving the issue. A recent investigation [36] proved the principle feasibility of target tracking in combination with RapidArc delivery. In absence of advanced methods, mid-ventilation could be applied as a first degree approach. Concerning management of (residual) breast movements mainly due to respiration, skin flash tools, aiming to expand beam fluence outside the body outline, have been proven and are normally used for IMRT treatments. In this study, no skin flash was applied, as mentioned in the methods, for two reasons: i) at planning level, the application of skin flash outside body outline has a minimal impact on the dose distribution since no dose is computed outside the body outline; ii) RapidArc, being based on different optimisation processes, does not generate a fluence map that can be ''expanded" outside the body to compensate for any effect. It is nevertheless obvious that, for treatment of real patients, it would be advisable to use both gated delivery and skin flash when normal IMRT is applied. For RapidArc, a work-around, to mimic skin flash, consists in the following process: i) generate two 3D CT dataset, one for dose calculation and one for plan optimisation; ii) expand the body in the optimisation CT dataset to artificially ''enlarge" the body outline, draw an enlarged target structure extending outside the original body outline, and perform optimisation on those wider body and target; iii) perform final dose calculation on the original CT dataset to account for the real size of the patient. This procedure has been tested for other patients and results technically feasible and could be considered as a first order, manual, substitute of the skin flash tool in RapidArc.

RapidArc was investigated for synchronous bilateral breast cancer and compared to fixed beam IMRT. RapidArc produced plans of high quality. Pre-treatment quality assurance showed reliability and high degree of agreement between calculated and delivered doses for both IMRT and RapidArc. RapidArc reduced treatment time of ~74%. The potential benefit of a better physical dose distribution, combined with a shorter delivery time makes RapidArc of interest also in the breast case, particularly in the perspective of target tracking.

## Competing interests

LC acts as Scientific Advisor to Varian Medical Systems and is Head of Research and Technological Development to Oncology Institute of Southern Switzerland, IOSI, Bellinzona.

## Authors' contributions

AF and LC designed the study. LC performed RapidArc and IMRT planning. GN, AC and EV performed measurements and data analysis. All contributed to writing, reviewing and approval of the manuscript.
